# Analysis of hazard perception characteristics based on driving behavior considering overt and covert hazard scenarios

**DOI:** 10.1371/journal.pone.0266126

**Published:** 2022-04-01

**Authors:** Tianzheng Wei, Tong Zhu, Chenxin Li, Haoxue Liu

**Affiliations:** 1 School of Automobile, Chang’an University, Xi’an, China; 2 College of Transportation Engineering, Chang’an University, Xi’an, China; 3 Patent Examination Cooperation Sichuan Center of the Patent Office, CNIPA Sichuan Chengdu, Chengdu, China; Tsinghua University, CHINA

## Abstract

The drivers’ hazard perception plays an important role in preventing and reducing the occurrence of traffic accidents. In order to explore the drivers’ hazard perception and their behavioral characteristics in overt and covert hazards, hazardous events of three traffic conflict types (vehicle to vehicle, vehicle to cyclist and vehicle to pedestrian) were designed for overt and covert hazards based on the UC-win/Road driving simulation software, respectively. 35 drivers were organized to conduct the driving simulation tests. The data of driving behavior was collected when they were driving. A comparative analysis of drivers’ hazard perception ability and driving behavior characteristics was carried out for hazardous scenarios and traffic conflict types. The result has shown that drivers are more likely to take slowing measures or brake earlier in overt hazard scenarios to ensure safe driving. And drivers are more likely to be involved in collisions in covert hazard scenarios. The types of traffic conflict have a significant effect on the hazard perception ability of drivers (F = 5.92, *p* < 0.01). Drivers have the strongest hazard perception for cyclists and the weakest hazard perception for pedestrians. Traffic conflict types has a significant effect on drivers’ average braking depth (F = 32.31, *p* < 0.01), average speed (F = 13.78, *p* < 0.01), and average acceleration (F = 9.26, *p* < 0.01).

## Introduction

Driver’s unsafe behavior is the main cause of road traffic accidents [1]. The driver is not only the user of the road, the manipulator of the vehicle, but also the feelers and feedback of the traffic environment in the road traffic system. Drivers should have the awareness of safety and risk-avoiding skills for driving safety. Defensive driving is a concept of safe driving that makes drivers stay away from the crash risk in advance. Prevention is the core of defensive driving. And hazard perception is an important component of driving skills, which is the premise and basis of prevention. Some study indicates that hazard perception is the ability to detect the potential hazards in the traffic environment [2, 3]. The accuracy of the driver’s hazard perception directly affects the driver’s judgment and decision in the traffic environment [4]. What’s more, uncertainty is a key problem of hazard perception [5]. Drivers’ misjudgment of potential hazards is the main cause of most traffic accidents [6]. It has been shown that hazard perception skills are the singly driving skills that are closely related to traffic accidents [7]. Therefore, improving drivers’ hazard perception of potential traffic risk is an important way to reduce traffic accidents [8].

The ability of drivers to perceive potential hazards in traffic is different, and the complex traffic environment would interfere with the accuracy of the driver’s perception of traffic hazards [9]. Vlakveld et al. [10] classified potential road hazards into overt hazards and covert hazards. Overt hazard means that the appearance of the hazard and its development process is completely visible. And covert hazard refers to the appearance of the hazard and its development process is partially hidden or completely hidden. In other words, the hazard is hidden to some extent. The driver’s hazard perception reflects the driver’s ability to make accurate predictions about the traffic risk and avoid it. The stronger hazard perception ability of the driver, the more possibility that the driver could avoid traffic collisions [11]. Research has shown that the driver’s hazard perception consists of sensation and cognition [12]. Sensation means that the driver finds a potential obstacle in the environment, but whether to interpret it as a hazard is uncertain. So the driver is required to find out the obstacle and identify whether there is really a hazard, i.e., further cognition. However, the driver’s perception of the hazard goes further than that; the driver’s reaction to the perceived hazard should also be taken into account. In other words, a driver is considered to have perceived a hazard if he or she reacts to it in some way.

Crundall et al. [13] pointed out that the hazard perception refers to the process in which drivers perceive information in the traffic environment and take reaction to it. Situational experimental methods and questionnaire assessment methods are often used to test drivers’ hazard perception abilities. Questionnaire methods mostly measure drivers’ hazard perceptions by surveying their subjective ratings of typical hazard scenarios or based on scales [14, 15]. Although the questionnaire method has many advantages for studying drivers’ hazard perception ability, the questionnaire results are easily influenced by drivers’ subjective consciousness. And people are often too optimistic when evaluating their driving ability and accident risk [16]. So, whether the questionnaire judged by drivers’ self-assessment or experience truly reflects the causes of certain road traffic accidents deserves further exploration. Situational experiments are used to test drivers’ hazard perception by real dynamic traffic scenarios. The drivers are usually asked to watch video clips or pictures containing dangerous events and to press the corresponding button as soon as they feel danger in the experiment. The driver’s hazard perception ability is judged by how fast or slow they feel danger [17–19]. Sagberg et al. [20] recruited drivers with different driving experiences to collect drivers’ reaction times by taking a video-based hazard perception test. The study showed that drivers’ average reaction times to hazard scenarios decreased with increasing experience. Finn et al. [21] tested drivers’ hazard perception based on pictures and videos of hazardous scenarios and got the result that the proportion of crashes among younger male drivers is high. One explanation was that they failed to perceive risk situations.

Simulated driving technology is also gradually being applied to test the risk perception ability of drivers with the development of virtual reality technology. On the one hand, Pradhan et al. [22] combined driving simulation and eye-movement techniques to explore the drivers’ hazard perception with different experiences through the variability of eye-movement parameters. Borowsky et al. [23] explored the effects of age and driving experience on drivers’ hazard perception through a situational experiment. On the other hand, the studies have shown that drivers’ hazard perception ability is positively correlated with safe driving behavior [24]. For instance, Warshawsky et al. [25] tested driver’s brake reaction and movement time based on the driving simulation tests. Meanwhile, driving behavior depends on the driver’s risk perception, driving habits and reaction characteristics [26]. Therefore, a single indicator of driving behavior may not reflect the hazard perception characteristics of drivers well.

Previous studies on drivers’ hazard perception often used indicators such as drivers’ reaction time and misjudgment rate to evaluate drivers’ hazard perception ability. There are few studies on the differences of drivers’ hazard perceptions when confronting different traffic conflict types and the impact of different potential traffic conflict scenarios on driving behavior. And studies have shown that the hazard perception of drivers in different regions is different [27]. Therefore, it is necessary to study the hazard perception ability and driving behavior of Chinese drivers when confronting different traffic conflict types.

The purpose of this study is to investigate the hazard perception ability of Chinese drivers in hazard events and their behavioral characteristics. To compare and analyze the driving behavior and hazard perception ability of Chinese drivers in the overt and covert hazard scenarios, three types of overt and covert hazard events were constructed based on the driving simulator. A total of 35 drivers were organized to collect the driving behavior in the hazard scenarios. The results of the study can be used in driver hazard testing and training to measure and improve drivers’ hazard perception awareness and defensive driving skills.

## Method

### Apparatus

**Fig 1:**
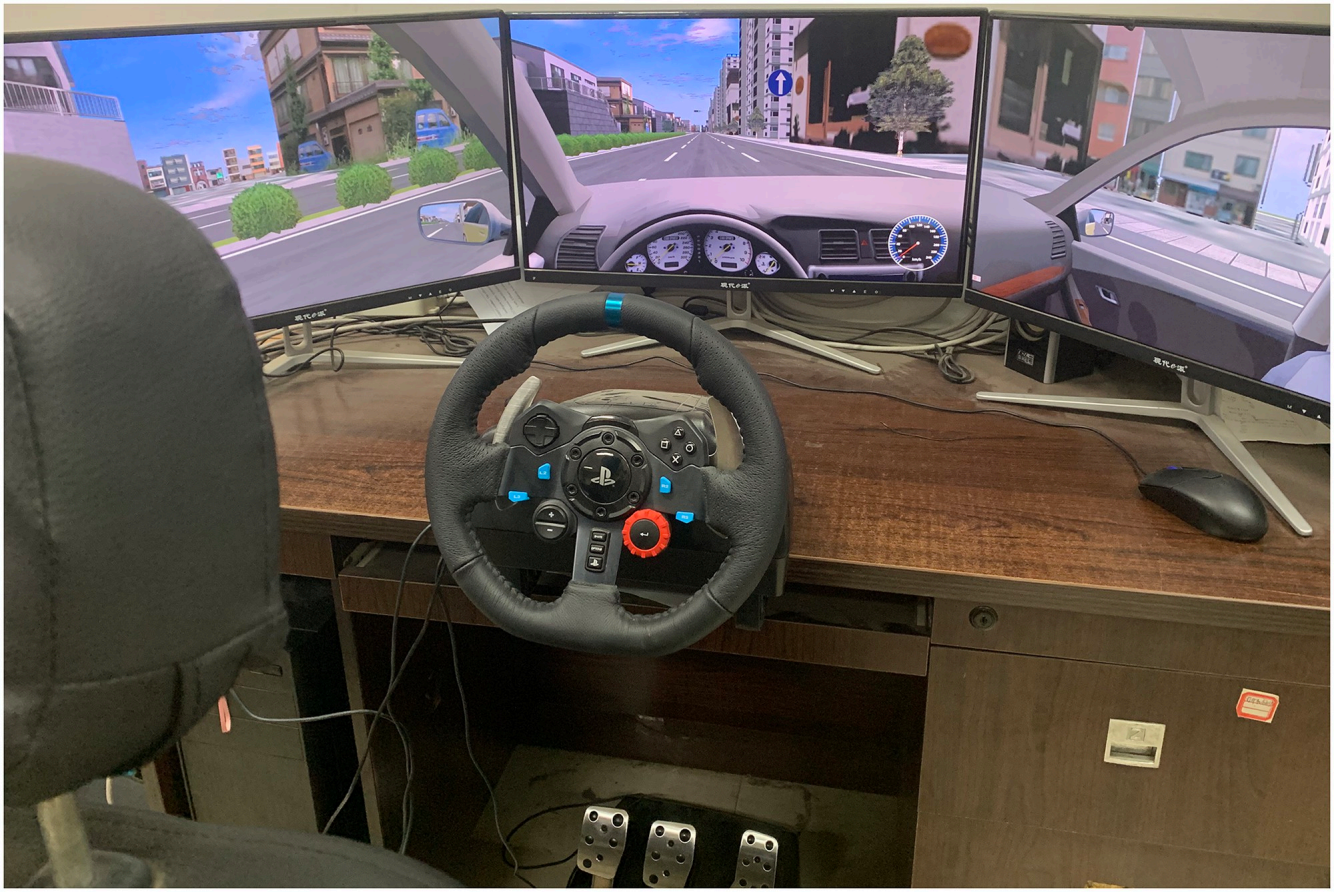
Driving simulator.

In this study, the driving simulator was mainly composed of driving simulation software and hardware equipment. the driving simulation software is UC-win/Road by FORUM8 company in Japan as shown in Fig 1. The scenarios were built by the software according to the user’s requirements. In addition, UC-win/Road also provided a data collection plug-in at the frequency of 25Hz during simulated driving to collect a series of data such as vehicle speed, acceleration, throttle, brake, steering velocity, etc. The hardware equipment consists of three parts, which were the scene display, the driving maneuvering system and the audio equipment. The scene display was mainly composed of three pieces of 24-inch LCD screens with 1920*1080 pixels. And the angle between two adjacent displays was 135°, mainly used to present the simulation driving scenarios. The driving maneuvering system mainly consists of a steering wheel with force feedback, accelerator pedal, brake pedal, clutch pedal, and gear lever, through which the driver could take the control of the vehicle in the simulated scenarios. The audio equipment was used to simulate engine sound, braking sound and the noise of the surrounding traffic environment while driving.

### Driving simulation experiment design

Combined with the infrastructure design of urban roads in Xi’an, Shaanxi Province to build a virtual simulation of urban roads scenarios. The urban road consists of five long straight two-way six-lane roads, which were connected by intersections. According to the Urban Road Engineering Design Specification [28], the road speed limit was 60 km/h. The signs and markings were set up according to the Urban Road Traffic Signs and Markings Setting Specification [29]. Trees, traffic lights and housing buildings were set on both sides of the road. In addition, warning signs for pedestrians and warning signs for construction were set at 50 m before the crosswalk and construction area. The ego vehicle was an ordinary medium-sized car. The driver was located in the middle lane with an initial speed of 0 km/h at the beginning of the experiment. A traffic flow of 500 pcu/h was set in the opposite lane.

This study chooses the usual types of traffic conflicts on urban roads [23, 30–35]. The driver’s hazard perception of road traffic scenarios was divided into the overt hazard and covert hazard, which were then divided into three traffic conflict events according to the driver’s conflict with other road participants: including the subject vehicle to vehicle conflict events (V-V), the subject vehicle to cyclist conflict events (V-C) and the subject vehicle to pedestrian conflict events (V-P). Hazardous events or action trajectory transformations were triggered by trigger points. A total of eight events were set up in this study. That includes two disturbance events, which can prevent drivers from being overly sensitive to changes in road traffic conditions and reacting inappropriately. The description of the hazardous events was shown in Table 1.

**Table 1:** Description of hazardous events.

Number	Potential road hazards	The type of events	Description
1	Disturbance event	V-V	The subject vehicle was located in the middle lane, and there was a vehicle driving in the same direction in front of its right lane.
2	Disturbance event	V-P	When the subject vehicle was approaching the intersection, pedestrians were walking along the direction of the subject on the right.
3	Overt hazard	V-V	The subject vehicle was located in the middle lane, and another car was moving forward in front of it in the right lane. The car changed its way to the middle lane after turning on the left turn signal light 3 seconds.
4	Covert hazard	V-V	The subject vehicle was driving in the outermost lane, the signal light was green in the straight direction, a work zone was blocked by the isolation fence in front of the intersection, a car from behind the isolation fence drove out.
5	Overt hazard	V-P	The subject vehicle was approaching the intersection when the signal light was green in the straight-ahead direction and a pedestrian rushes into the crosswalk.
6	Covert hazard	V-P	The subject vehicle was driving in the middle lane, the pedestrians in front of it hidden by trees in the median separator. As the subject vehicle approaches the crosswalk, a pedestrian suddenly enters the crosswalk.
7	Overt hazard	V-C	The subject vehicle was traveling in the middle lane with a cyclist in front of its right lane, whose forward direction was blocked by the work zone, and the cyclist changed its way to the middle lane.
8	Covert hazard	V-C	The subject vehicle in the right lane wants to go straight through the intersection, at this time the signal light in the straight direction was green. At the intersection in the right front of the vehicle, a bus parked next to the intersection waiting for the green light, a cyclist behind the bus suddenly rushed out.

### Procedure

The experimenter guided the subjects to fill out a personal information questionnaire before the experiment, which included information about the driver’s name, gender, driving experience and age. After the subjects filled out the questionnaire, the experimenter guided the driver to the driving simulator to prepare for the driving simulation test. After the subjects were prepared, the experimenter guided them to practice the driving simulation in the practice scenarios until they were proficient in the driving simulation. After a rest of about 5 minutes, the subjects were guided to start the formal driving simulation test. The subjects were required to strictly follow the traffic rules and test guidelines during the formal experiment. What’s more, it was not permitted for drivers to drive vehicles into collisions with any traffic participants or road infrastructure while driving. Once a collision occurs, the sound system will make a collision sound immediately. And the red text "collision!" appeared in the middle of the display screen at the same time. The vehicle will not be destroyed and the driver could continue to complete the driving tasks after the collision.

### Subjects

A total of 35 drivers (25 men and 10 female) with valid C1 licenses was randomly recruited. The driver’s age ranged from 21 to32 years old ($\overset{-}{x}=24.86,\;s=2.12$) and the driving experience distributed from 1 to 9 years ($\overset{-}{x}=3.63,\;s=1.86$). All drivers’ driving experience was counted from the time of obtaining a driver’s license. The drivers has no less than one year of driving experience and has real driving experience. The driver was required with a normal vision or normal after correction of vision, no physical disease and no alcohol or drug consumption within 24 hours before the experiment.

### Hazard perception indicators

**Fig 2:**
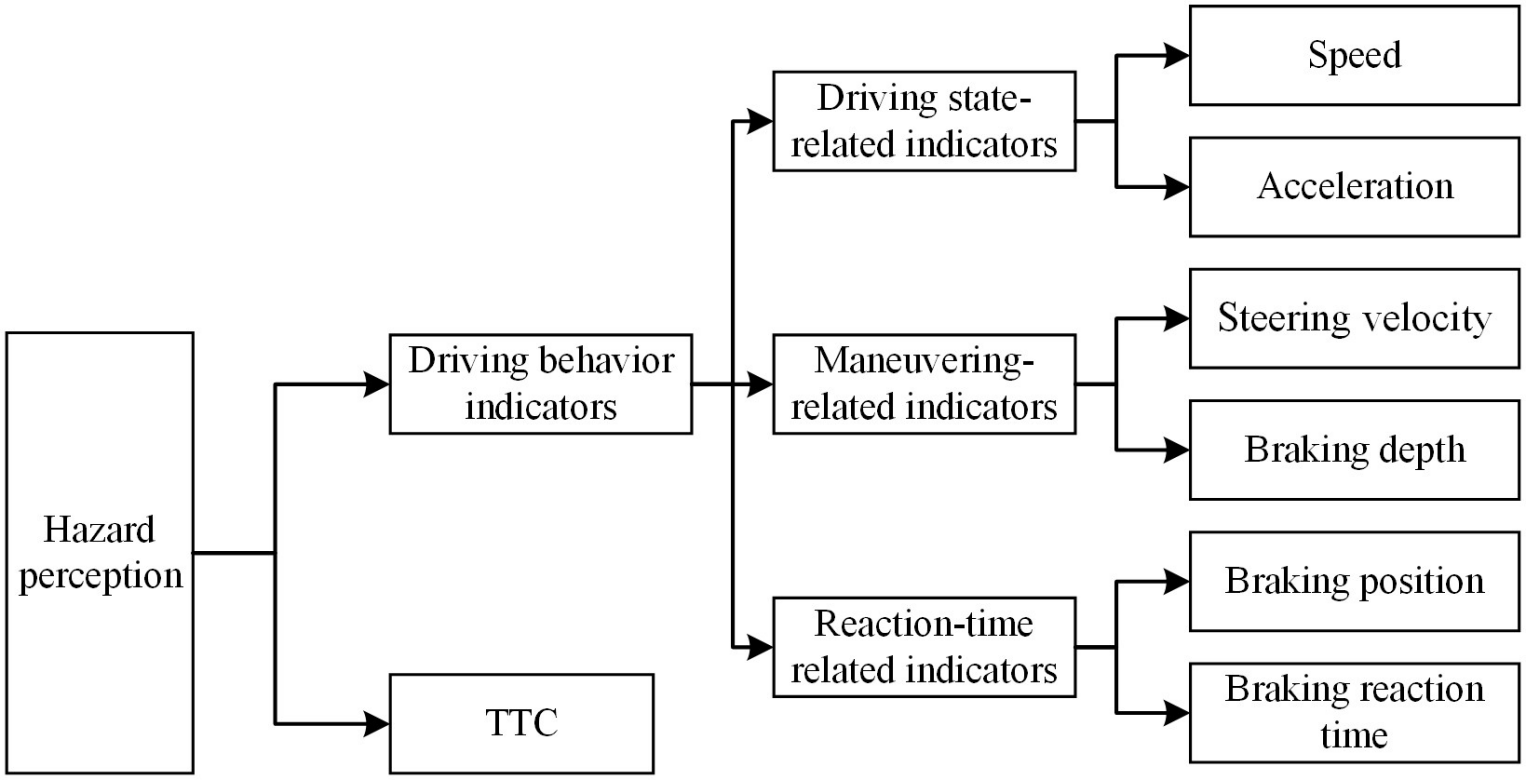
Hazard perception indicators.

In order to represent the driver’s hazard perception as comprehensively as possible, the driver’s driving behavior data were collected 150 m ahead of the point location of the expected collision [36]. And the indicator is further divided into driving state-related indicators, maneuvering-related indicators and reaction-time related indicators (Fig 2). The driving state-related indicators include the average speed and average longitudinal acceleration of the vehicle. These two indicators could reflect the overall trend of the driver’s response in hazard scenarios. Maneuvering-related indicators include the steering velocity and the average brake depth. The steering velocity is the rate of change of the steering wheel cornering. Brake depth indicates the ratio of the driver’s brake pedal force input value to the maximum braking force. And the brake depth was represented by 0 to 1, where 0 indicates no brake input and 1 indicates full braking.

**Fig 3:**
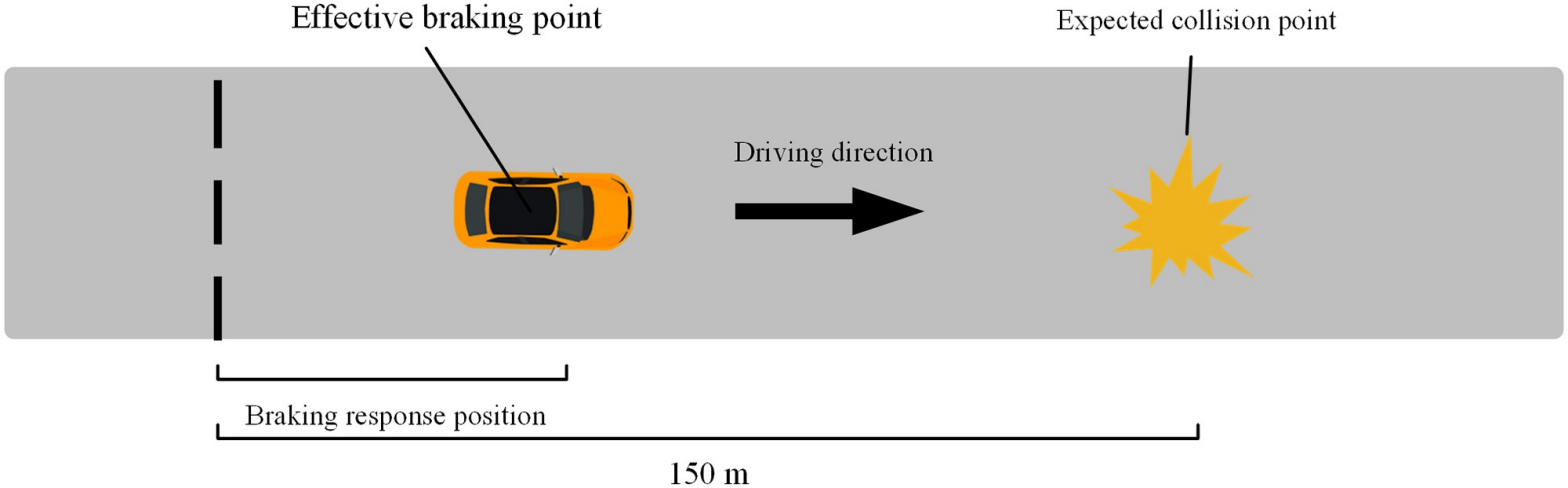
The brake reaction position.

The reaction-time related indicators could demonstrate the differences in driver hazard perception characteristics from a microscopic perspective. And the braking position and braking reaction time normalized were selected as the reaction-time related indicators in this study. The braking depth greater than 5% of the maximum braking depth was regarded as an effective brake [36]. The braking position was determined by the difference between the position where the hazard scenario starts and the position where the driver made an effective braking response (Fig 3). The interval between the start time of the hazard scenario and the time when the driver made an effective braking reaction is the braking reaction time. The driver’s driving time in the hazard scenario was the interval between the start time of the hazard scenarios and the time of the driver driving away from the position of the expected collision conflict point. Considering the variation of the driver’s speed resulting in different times in each scenario, the braking reaction time was normalized. And the normalized braking reaction time was equal to the ratio of the braking reaction time to the total time spent by the driver driving in the hazard scenario. The common traffic conflict indicator that the time to collision (TTC) was selected as the evaluation indicator of driver’s hazard perception ability [37, 38]. The TTC value was calculated by Hayward [39]. In this study, the beginning moment of traffic conflict is regarded as the moment when the driver takes an effective brake in a hazard scenario. In other words, if the driver does not apply the brake at the moment of traffic conflict, the vehicle will travel at the current speed and will collide with the target object after a period of time.

At some point in the car following scenario, let’s the lead vehicle is in position *s*_*l*_, *v*_*l*_ is the speed of the lead vehicle. The following vehicle is in position *s*_*f*_. And *v*_*f*_ is the speed of the following vehicle. The relative distance between two vehicles is described as follows:

$$\Delta s=s_{l}-s_{f}$$ (1)

The relative speed between two vehicles is described as follows:

$$\Delta v=v_{f}-v_{l}$$ (2)

Thus, the TTC values are calculated as follows in each hazard scenario.

$$TTC=\frac{\Delta s}{\Delta v}$$ (3)

where, Δ*v*>0. The smaller the TTC value is, the worse the hazard perception of the driver is, and the more likely to be involved in a crash.

## Results

### Analysis of the hazard scenario’s emergency

The driver tends to take the emergency braking, jerk the steering wheel or both to avoid a crash when the driver perceives danger in the driving process. In this study, most of the subject drivers had a small steering velocity in the hazard scenarios. It was not possible to accurately describe the driver’s hazard avoiding response behavior. So the effective brake was used to evaluate the driver’s response in the hazard scenario. The effective brake was defined as a brake that braking depth is greater than 5% of the maximum braking depth according to the method of Ba [36]. The response rate of the driver was defined as the ratio of the number of those who made effective brakes to the total number of subjects in a hazard scenario. And the driver’s emergency response to a hazard scenario was obtained based on whether the driver made an effective brake.

**Fig 4:**
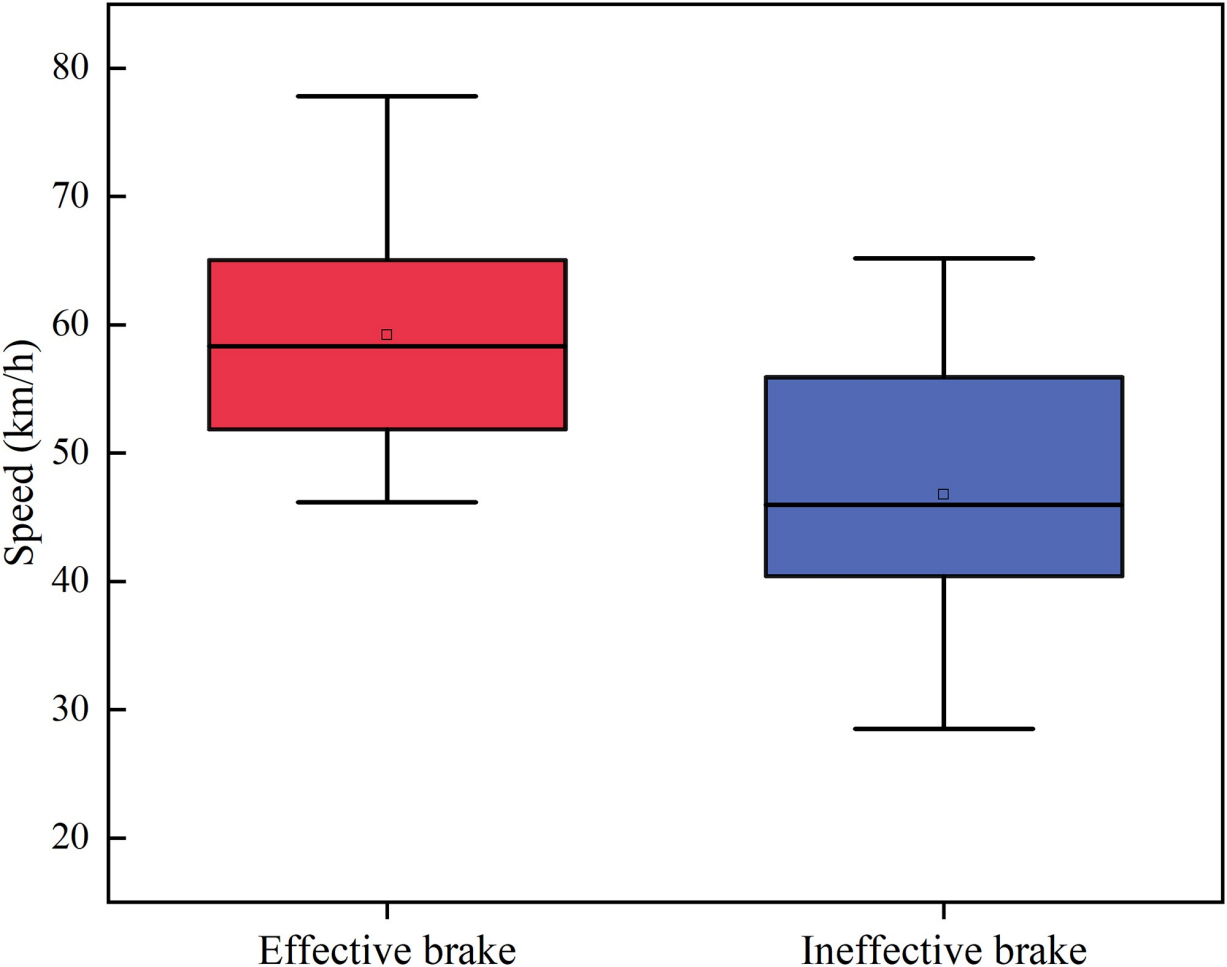
The subject vehicle’s average speed in the V-V events of overt hazard.

The results show that the average response rate of the driver for covert hazard scenarios is 85.7% and overt hazard scenarios is 66.7%. For the three types of traffic conflict events, the V-P events have the highest response rate (overt hazard 80%, covert hazard 94.28%). The response rate for the V-C events is second (overt hazard 71.43%, covert hazard 91.43%). And the overt hazard scenarios of the V-V events have a lower response rate for the driver (45.7%) than the covert hazard (71.4%). To further investigate the reasons for the lower effective braking response rate of drivers in V-V events of overt hazard, the speed of the subject vehicle was extracted when the driver makes an effective brake in this event. And the speed of the vehicle at the trigger point of this hazard event was extracted for drivers who did not take an effective brake. The driver’s speed in the V-V events of overt hazard is shown in Fig 4. The descriptive statistical analysis shows that the average speed of drivers who did not take effective brake is 46.3 km/h, while the drivers who took effective brake is 59.2 km/h. Based on the analysis of the average speed of the overt hazard and covert hazard scenarios, the average speed of drivers in the overt hazard scenarios (44.28km/h, SD = 9.20) is lower than that of the covert hazard scenarios (45.87km/h, SD = 11.09). It can be seen that drivers focus on speed control and drive their vehicles at a lower speed in V-V events of overt hazard.

### Analysis of driving state-related indicators

It was found that most drivers took very little change in the lateral position of the vehicle in the hazard scenarios through the analysis of the driver’s driving state-related indicators. Therefore, the driving state-related indicators of this study were analyzed using the average speed and average longitudinal acceleration of the driver in the hazard scenarios. When the driver encounters a dangerous situation, the lower vehicle speed could ensure that the driver has enough time to take measures to offset the risks. Therefore, whether the driver slows down and maintains a low speed is an important evaluation indicator of the driver’s hazard perception in a dangerous driving situation. Multi-factor analysis of variance conducted on the hazard scenarios types, traffic conflict types and average speed yielded that traffic conflict types have a significant effect on driver’s average speed (F = 13.78, *p* < 0.01). The results of the pairwise comparisons are shown in Table 2, where drivers have the highest average speed in the V-V events. And drivers have the lowest average speed in the V-P events.

**Table 2:** Pairwise comparison of average speed.

Traffic conflict type		Mean difference (I-J)	SE	Sig.	95% confidence interval of the difference	
(I)	(J)				Lower limit	Upper limit
V-V	V-P	8.674	1.928	.000	4.864	12.483
V-P	V-C	-7.752	1.760	.000	-11.228	-4.275
V-C	V-V	-.922	1.959	.639	-4.792	2.948

Note: SE refers to the standard error. Sig refers to the *p*-value.

**Fig 5:**
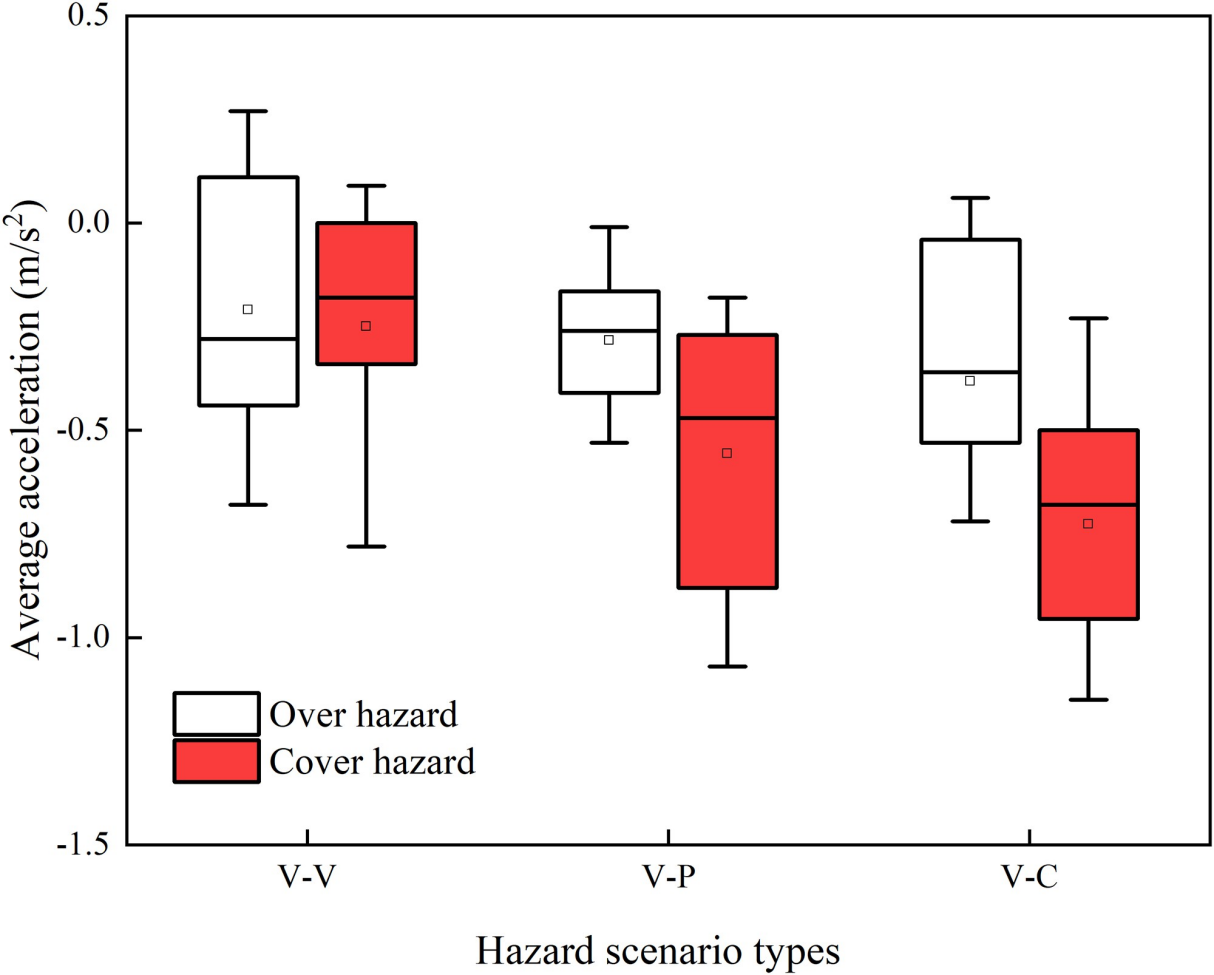
The subject vehicle’s average acceleration in the hazardous scenarios. The meaning of the negative value of the vertical axis is deceleration. And the meaning of the positive value is acceleration.

The vehicle’s longitudinal acceleration is also an important indicator for evaluating the hazard perception of the driver. The smaller vehicle longitudinal acceleration indicates that the driver’s driving process is relatively smooth, which reflects the driver’s correct prediction and judgment of the surrounding traffic environment and the effective control of the vehicle in the dangerous scenario to a certain extent. Multi-factor analysis of variance was performed on hazard scenario types, traffic conflict types and driver average acceleration. The results show a significant difference in the effect of hazard scenario types on driver acceleration (F = 13.69, *p* < 0.01), with the average driver acceleration in the overt hazard scenarios being 0.2 m/s^2^ smaller than the covert hazard. The 95% confidence interval is 0.102–0.336 m/s^2^. Traffic conflict types have a significant effect on driver average acceleration (F = 9.26, *p* < 0.01). The interaction between hazard scenario types and traffic conflict types has no significant effect on driver average acceleration (F = 2.16, *p* = 0.12). The results of the pairwise comparisons are shown in Table 3. It can be concluded that drivers have greater acceleration in the covert hazard scenarios and that drivers in the V-C events take the greatest acceleration among the three types of traffic conflict events as shown in Fig 5.

**Table 3:** Average acceleration pairwise comparison.

Traffic conflict type		Mean difference (I-J)	SE	Sig.	95% confidence interval of the difference	
(I)	(J)				Lower limit	Upper limit
V-V	V-P	.190	0.074	0.011	0.044	0.337
V-V	V-C	.324	0.075	0.000	0.175	0.473
V-P	V-C	.134	0.068	0.049	0.000	0.268

### Analysis of maneuvering-related indicators

Drivers who could correctly anticipate possible hazards will usually avoid them by gentle braking or gentle lane change maneuvers when driving in a hazardous scenario. However, if the hazard perception ability of the driver is poor or the driver makes a wrong prediction of the danger, drivers often use emergency braking or steering wheel to avoid collision when danger appears. Therefore, the braking and steering operations of drivers can reflect the difference in drivers’ ability to perceive hazards in dangerous scenarios. Multi-factor analysis of variance of steering velocity for drivers in different hazard scenarios and traffic conflict types, it is found that no significant differences in steering velocity for drivers in overt and covert hazard scenarios (F = 0.08, *p* = 0.78) and different types of traffic conflicts (F = 0.18, *p* = 0.84). The interaction of hazard scenarios and types of traffic conflicts has no significant effect on steering wheel rotation rate (F = 0.53, *p* = 0.59).

Multi-factor analysis of variance on the average braking depth of drivers in different hazard scenarios and types of traffic conflicts revealed that the types of traffic conflicts have a significant effect on the average braking depth of drivers (F = 32.31, *p* < 0.01), while the hazard scenarios and its interaction with the types of traffic conflicts have no significant effect on the average braking depth of drivers (F = 2.61, *p* = 0.08). The results of the pairwise comparisons are shown in Table 4, where drivers braked significantly deeper during the V-P events than the V-V and V-C events and have the lowest braking depth during the V-V events.

**Table 4:** Pairwise comparison of average braking depth.

Traffic conflict type		Mean difference (I-J)	SE	Sig.	95% confidence interval of the difference	
(I)	(J)				Lower limit	Upper limit
V-P	V-V	.086	.011	.000	.065	.108
V-P	V-C	.046	.010	.000	.027	.066
V-C	V-V	.040	.011	.000	.018	.062

### Analysis of reaction-time related indicators

Reaction-time related indicators can reflect drivers’ perception of hazard from a more intuitive perspective. In general, it is indicated that when the braking reaction time is short and the braking reaction position of the driver is far away from the traffic participants of danger, the driver perceives the potential danger encountered in the driving environment earlier. The long braking reaction time and the proximity of the braking reaction position to the traffic participants of danger indicate that the driver’s perception of potential road hazards in the traffic environment is inadequate. In the event of a dangerous situation, more drastic measures will be taken in response.

The results of multi-factor analysis of variance on the driver’s braking position in different hazard scenarios and traffic conflict types shown that the types of traffic conflicts (F = 0.92, *p* = 0.40), hazard scenarios (F = 0.35, *p* = 0.56) and their interaction (F = 3.01, *p* > 0.05) has no significant effect on the driver’s braking position. The mean value of the driver’s braking position in the covert hazard scenarios (81.03 m) is greater than that in the overt hazard scenarios (78.89 m). It indicates that the driver’s position is closer to the point of expected conflict when taking the brake in the covert hazard scenario. Descriptive statistical analysis revealed that among the traffic conflict types, drivers have the smallest average braking position in the V-C events (73.87 m), and the V-P events have the second-lowest average braking position (81.25 m). Similarly, the results of the multi-factor analysis of variance showed that hazard scenarios, types of traffic conflicts and their interaction has no significant effect on driver braking reaction time (*p* > 0.05). The descriptive statistical analysis shows that the braking reaction time of the driver in the covert hazard scenarios is smaller than that in the overt hazard scenarios. The braking reaction time for V-C events is the smallest, followed by V-P events, and drivers have the largest braking reaction time for V-V events.

### Analysis of driver’s hazard perception

**Fig 6:**
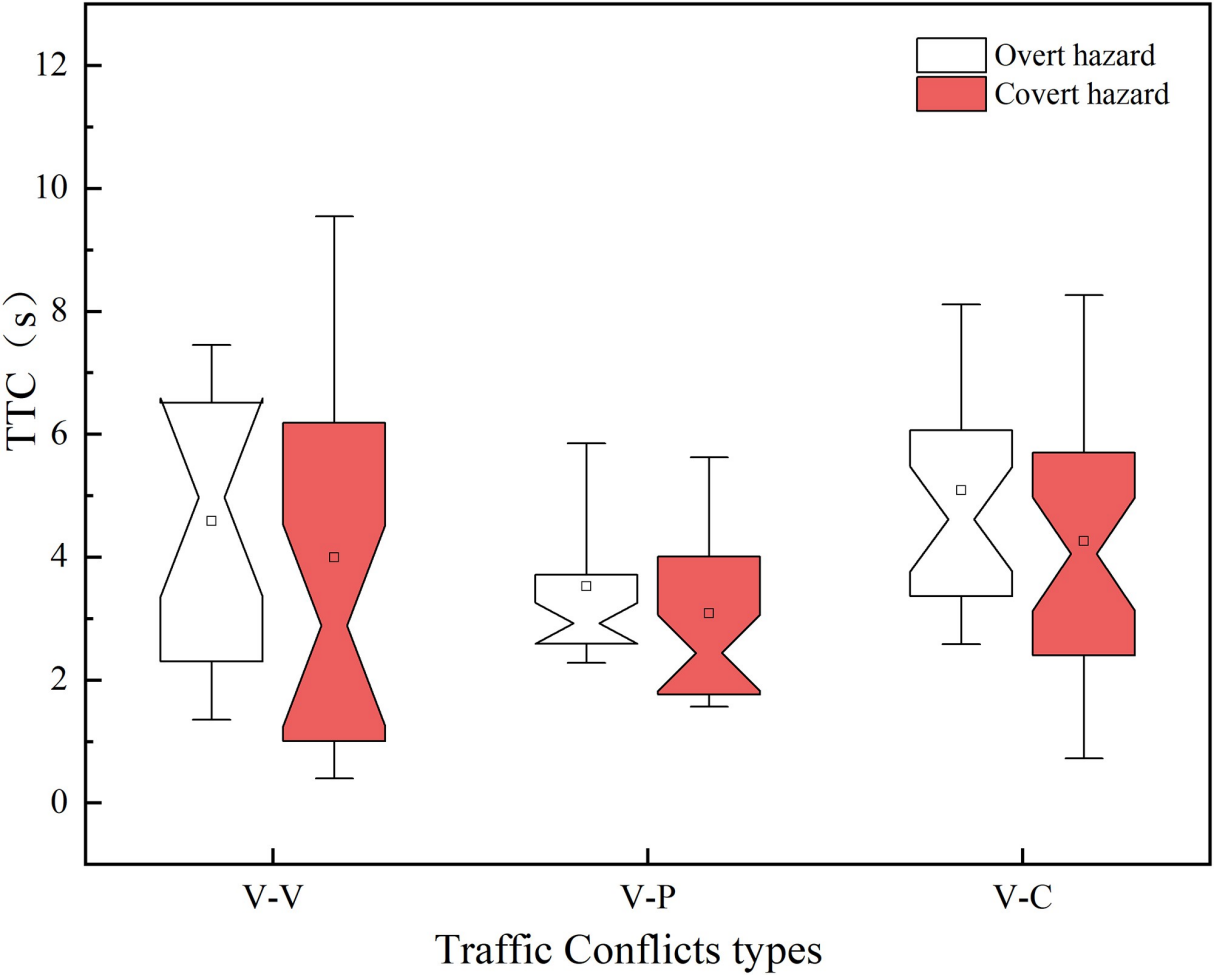
TTC of three types of traffic conflict events.

In this study, we used the TTC calculation method defined by Hayward [39] and used the TTC value as an evaluation indicator for the severity of traffic conflict events [40, 41]. As shown in Table 5, the median TTC value (Me) for the overt hazard scenarios is 3.52s and the mean value is 4.14s, both of which are higher than the covert hazard scenarios (Me = 2.90s, Mean = 3.60s). Combined with the TTC values of the three traffic conflict events shown in Fig 6, it can be concluded that among the three traffic conflict types, drivers are more likely to be involved in collisions in the covert hazard scenarios than in the overt hazard scenarios.

**Table 5:** TTC values in hazard scenarios.

Hazard scenarios type	Mean	SD	Me	25% percentile	75% percentile
Overt hazard	4.14	1.80	3.52	2.60	5.68
Covert hazard	3.60	2.47	2.90	1.64	5.47

To explore the effect of traffic conflict types, hazard scenarios and their interaction on TTC, the results of the multi-factor analysis of variance show that traffic conflict types have a significant effect on TTC (F = 5.92, *p* < 0.01). The pairwise comparisons of traffic conflict types results are shown in Table 6, where V-P events have the lowest TTC values and take a higher crash risk.

**Table 6:** TTC of pairwise comparison.

Traffic conflict type		Mean difference (I-J)	SE	Sig.	95% confidence interval of the difference	
(I)	(J)				Lower limit	Upper limit
V-V	V-P	.400	.164	.016	.076	.723
V-P	V-C	-.485	.149	.001	-.780	-.190
V-C	V-V	.086	.166	.608	-.243	.414

Jin et al [42] used the TTC values of 3s and 5.5s as two thresholds for the warning system to classify driving safety into three levels. In this study, we took the same approach and classified the crash risk into low risk, medium risk and high risk. TTC ≥ 5.5s indicates that the crash risk is low and the driver has a high hazard perception ability. 3.0 ≤ TTC < 5.5s indicates that the crash risk is medium and the driver’s hazard perception ability level is medium. TTC < 3.0s indicates that the crash risk is high and the driver’s hazard perception ability is weak.

The effects of driver’s age, gender and driving experience on driver hazard perception ability were investigated separately. ANOVA analysis that gender has no significant effect on driver crash risk (F = 0.59, *p* = 0.44). And driving experience has a significant effect on hazard perception ability (F = 4.27, *p* < 0.01). Age has a significant effect on driver crash risk (F = 2.11, *p* = 0.04 < 0.05).

**Fig 7:**
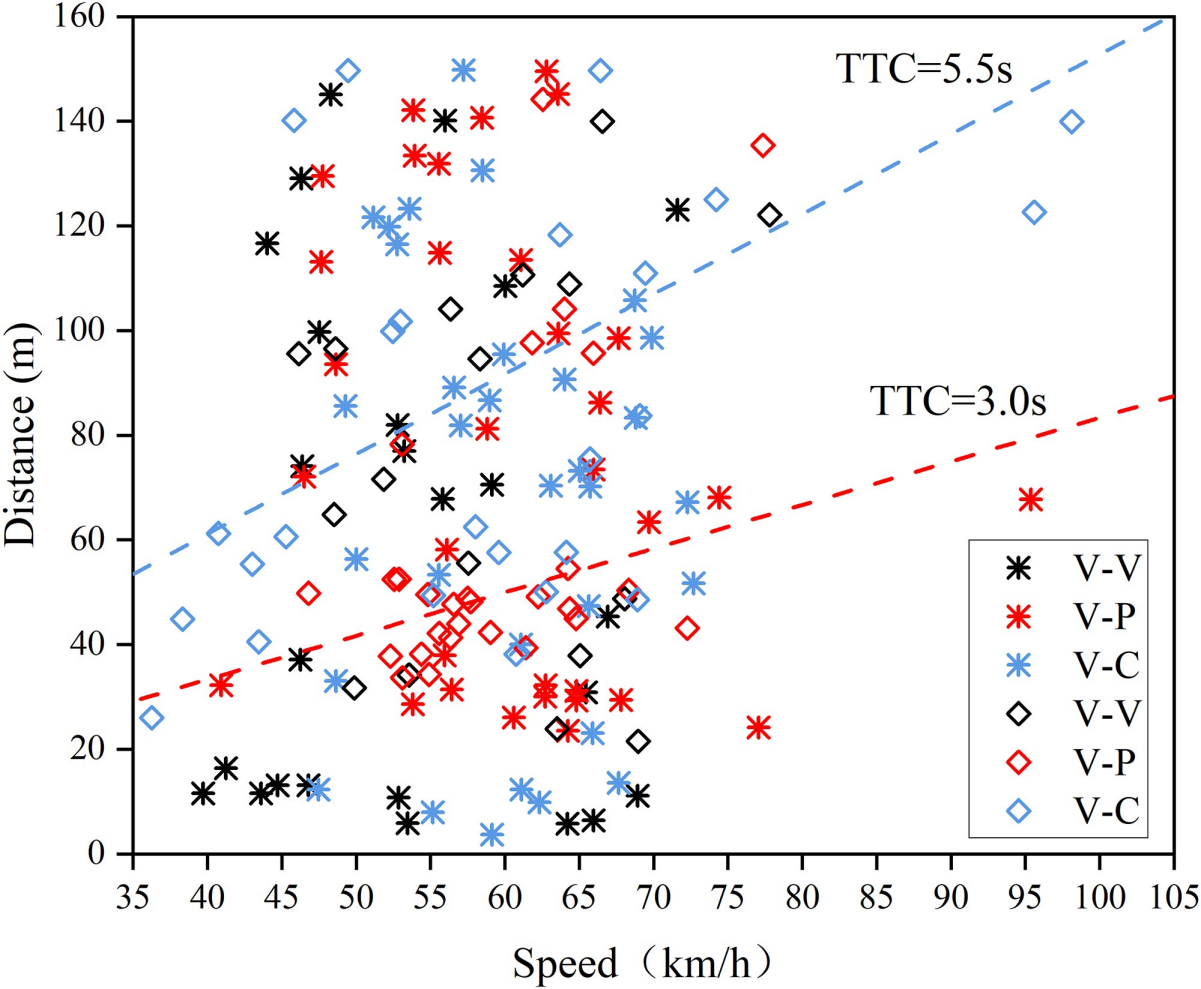
Vehicle speed-distance distribution in hazard scenarios (* indicates the covert hazard. ◇ indicates the over hazard).

Fig 7 shows the scatter plot of the crash risk in the hazard scenarios. The abscissa is the instantaneous vehicle speed at the moment of braking response. The ordinate is the distance from the point of the expected crash to the point of braking response. In the overt hazard scenarios where the driver made an effective brake response, 24 cases (34%) have a TTC < 3.0s with high crash risk and 20 cases (28.6%) have a TTC ≥ 5.5s with low crash risk. There are 14 V-P events with high collision risk and the vehicle speed is distributed between 52.30–72.27 km/h. The distance from the point of the expected crash is distributed between 33.66–50.34 m with high safety risks. Overall, the driver’s speed distribution is more discrete. And drivers with high collision risk tend to control their speed around 60 km/h.

The braking distance distribution of each hazard scenario is more dispersed in the covert hazard scenarios (Fig 7), but the distribution of vehicle speed was more concentrated. Among all the covert hazard scenarios where drivers made effective braking, 37 cases (41.1%) with TTC < 3.0s are at high crash risk and 32 cases (35.5%) with TTC ≥ 5.5s are at low crash risk. 10 cases of high collision risk in the covert hazard scenarios originated from V-V events.

**Fig 8:**
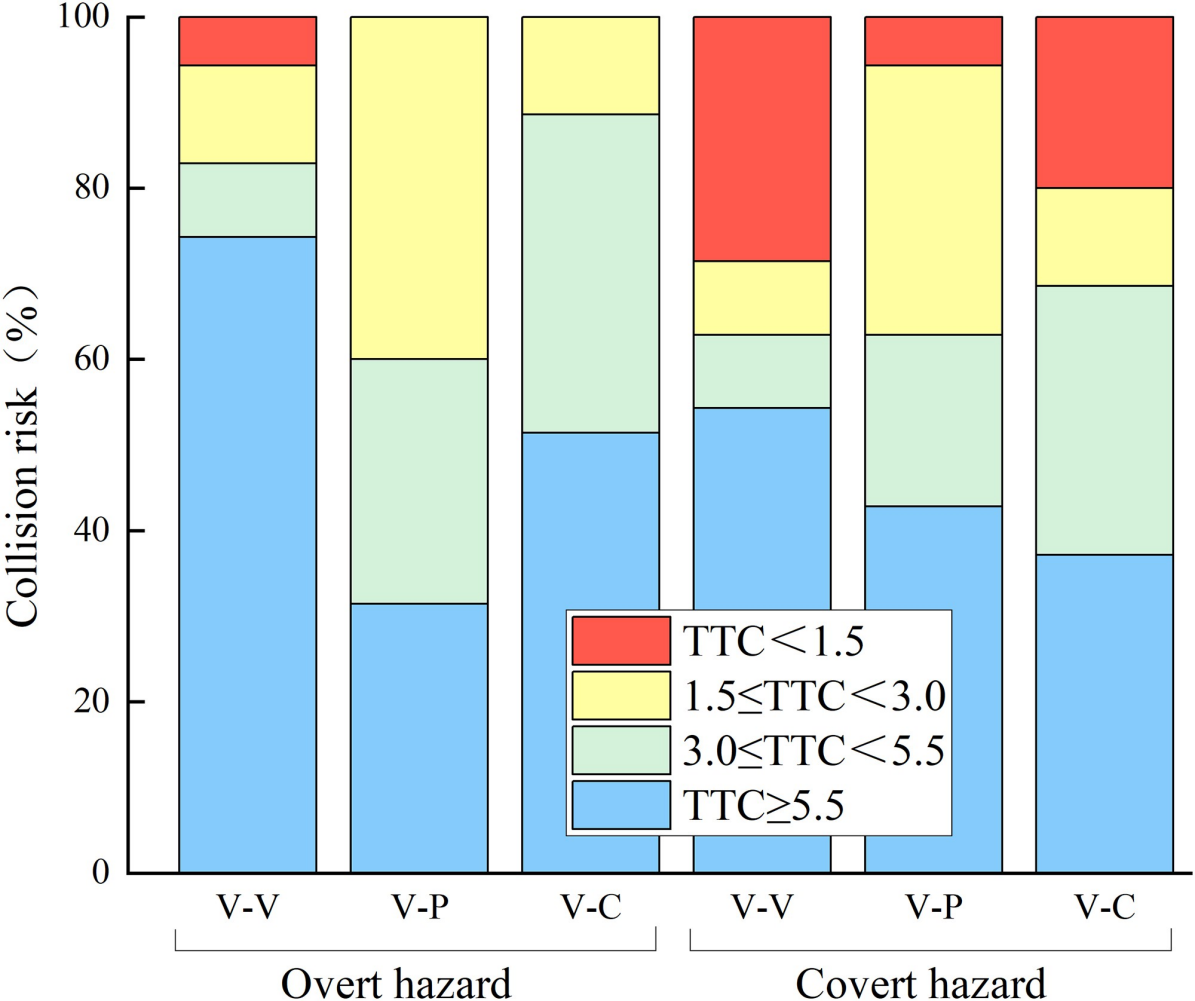
Percentage stacking bar chart of collision risk.

Further, influenced by drivers’ driving habits and personality factors, some drivers think that the collision conflict will not necessarily occur and take certain risk-averse behavioral measures that could avoid a collision when the TTC < 3.0s. To explore the likelihood of the collision in hazard scenarios, the research has concluded that a TTC less than the limit of 1~1.5s is an effective crash alternative [43]. In this study, 1.5s was used to be a limit instead of a crash [44]. Collision risk was classified into crash, high risk, medium risk and low risk. The percentage stacked bar charts were used to show the difference in crash risk between different hazard scenarios (Fig 8). It was found that the vehicle speed was generally low or has already slowed down in advance when the driver has no effective brake reaction. And vehicles could safely pass the hazard scenarios. We considered that the driver reacted before the hazard events occurred, so the driver who did not react with the brake was considered a low crash risk. Fig 8 shows that the highest percentage of crashes occurred in the V-V events, followed by the V-C events. Drivers have a lower percentage of collisions in the V-P events, although they took a higher risk of collision.

## Discussion

This study discusses the characteristics of drivers’ hazard perception behaviors for different participants in overt and covert hazard scenarios. The analysis provides some additional insights into drivers’ hazard perception. Firstly, drivers’ hazard avoiding behaviors in hazard scenarios can better reflect drivers’ hazard perception ability. Drivers’ driving behavior can better measure and distinguish drivers’ hazard perception ability in covert hazard scenarios. Drivers usually take measures in advance to prevent collisions in overt hazard scenarios. Drivers’ hazard perceptions are different for potential traffic conflicts when confronting different road participants. Drivers have poor hazard perception for pedestrians, followed by hazard perception for cyclists and the best hazard perception for vehicles in the overt hazard scenario. In the covert hazard scenario, drivers are less able to perceive the hazards of vehicles and pedestrians. Drivers have a higher likelihood of collision risk with vehicles and cyclists. These findings could provide an important reference for driver hazard perception testing and driver safety training.

### Effects of the overt and covert hazard scenarios on drivers’ hazard perception

The response of the driver to the covert hazards is more violent. In particular, the average speed of drivers is almost distributed in the interval of 30–60 km/h in this study. What’s more, the value of the average speed for the overt hazard scenarios is 44.28 km/h and that for the covert hazard scenarios is 45.87 km/h. However, drivers’ braking deceleration is significantly greater in the covert hazard scenarios than that in the overt scenarios (F = 13.69, *p* < 0.01). Further, at the moment of driver brake response, 45.7% of drivers in the overt hazard scenarios were overspeeding (with a speed limit of 60 km/h). And 43% of drivers were overspeeding in the covert hazard scenarios. It indicates that drivers perceive a higher risk in covert hazard scenarios [45], which is a greater threat to driving safety. Indeed, the number of cases of collisions in overt hazard scenarios (1.9%) is found to be less than that in covert hazard scenarios (18.1%) through the analysis of collision accidents. On the other hand, the driver’s braking deceleration in the covert hazard scenario is greater and the vehicle speed is higher. The driver taking a greater braking depth will result in a greater change in vehicle speed, which will cause greater harm to other vehicles in the traffic flow [46]. This is mainly related to the poor situational awareness of drivers [47], who are unable to perceive potential hazards in the traffic environment promptly. And the driving behavior of drivers in covert hazard scenarios can better reflect the driver’s hazard perception.

### Analysis of drivers’ hazard perception for different traffic conflict types

Drivers have different hazard perceptions for different traffic conflict events, with the weakest hazard perception for pedestrians and the strongest hazard perception for cyclists. The types of traffic conflict have a significant effect on the driver’s average speed, average acceleration, average braking depth and TTC. Among the three types of traffic conflict events, drivers have the lowest average speed (Table 2), the greatest average braking depth (Table 4) and the lowest percentage of collisions with pedestrians during V-P conflict events (Fig 8). It shows that drivers will try to avoid collision accidents with pedestrians in the traffic environment. The reason for the lower collision accidents for pedestrian hazard scenarios may be due to the traffic management regulations that motorists need to slow down when approaching crosswalks. At the same time, pedestrians move at a slower speed compared to cyclists and vehicles in the traffic environment, drivers often take a larger brake for deceleration operations to avoid collisions when pedestrians suddenly break into crosswalks.

The driver’s hazard perception of the cyclist is strong. The driver could easily predict in advance that the cyclist will change lanes ahead of the driver’s current lane in the overt hazard scenarios. So, the subject drivers often took deceleration early and have awareness of deceleration contingency. Although drivers would take braking measures as soon as possible to avoid collisions in the covert hazards, the likelihood of a collision was still higher than that in the V-P events. One possible reason is that the speed of cyclists is higher compared to pedestrians. The average speed of drivers is the highest in the V-V events. In the overt hazard scenarios of V-V events, 74.3% of drivers have high awareness and take measures in advance to pass the scenario with low crash risk. The reason for collisions in V-V events is mainly due to the driver’s high expectation that the vehicle will drive normally, thus causing it to be difficult for the subject driver to avoid a collision when the vehicle suddenly breaks into the subject driver’s lane. In the covert hazard scenario, the conflicting vehicle is hidden and the speed of the vehicle is high. If the driver cannot perceive the potential risk in advance and take measures, then there is a high risk of a collision. This may be the reason why drivers in the V-V covert hazard scenario is prone to collisions when they are at high risk of collision.

### The influence of driver characteristics on hazard perception

The effects of driver age, gender and driving experience on driver hazard perception were investigated separately to explore the variability of hazard perception ability among driver types. ANOVA analysis that gender has no significant effect on driver crash risk (F = 0.59, *p* = 0.44) and driving age has a significant effect on hazard perception ability (F = 4.27, *p* < 0.01). It has been shown that the drivers’ hazard perception ability is significantly enhanced as the driving experience increases [48]. And through the least significant difference method for multiple comparisons, the hazard perception of a collision is more sensitive and can detect the danger earlier with the increase in driving experience [49]. It has been shown that the experienced drivers can find more cues in the traffic environment. And the perception of them from a single dimension to an assessment of the overall riskiness of the traffic environment to perceive more potentially hazardous situations [50, 51]. Age has a significant effect on driver crash risk in this study (F = 2.11, *p* = 0.04 < 0.05) [52]. However, past research has also found that driver hazard perception is not affected by age [53]. Drivers’ reactions become slower as they get older, which may involve a difference between the hazard perception and motor ability [23]. The reason for the variability in this study may be due to the inclusion of drivers’ responses into consideration and the relatively narrow age distribution of the subject drivers. Therefore, the effect of age on drivers’ hazard perceptions still needs further research.

### Advantages and limitations

On the one hand, it is a better way to evaluate the driver’s hazard perception ability based on the cues found in potential hazard situations or behavioral responses to potential hazards. The study of drivers’ hazard perception for different road users in overt and covert hazards can better identify the comprehensive ability and perceived vulnerability of drivers to avoid traffic accidents. On the other hand, the study of driver’s hazard perception plays a crucial role in elucidating the mechanism of traffic accidents. It also provides a theoretical basis and methods for unsafe driving behavior intervention and driver training.

In this study, there are fewer types of hazardous scenarios and drivers only have traffic conflict risks with a single traffic participant in one hazardous scenario. However, drivers may be at risk of traffic conflicts with multiple traffic participants at the same time in real-world traffic environments. On the other hand, the minimum TTC value can be adopted as an alternative assessment index for crash risk, and the driver’s perception of danger can be measured more accurately with the driver’s eye movement data.

## Conclusions

In this study, the behavioral characteristics of drivers’ hazard perception ability under overt and covert hazards were explored based on the driving simulations. A comparative analysis of drivers’ hazard perception ability and driving behavior between vehicles, pedestrians and cyclists in hazard scenarios was conducted. The conclusions are as follows: Drivers’ hazard perception ability for different traffic conflict types are significantly different (F = 5.92, *p* < 0.01). The strongest hazard perception ability is for cyclists and the weakest hazard perception ability is for pedestrians. The driver is more likely to be involved in collisions in covert hazard scenarios than in overt hazard scenarios. Drivers’ perception of hazard urgency is reflected in driving behavior. And traffic conflict types have a significant effect on drivers’ average braking depth (F = 32.31, *p* < 0.01), average speed (F = 13.78, *p* < 0.01), and average acceleration (F = 9.26, *p* < 0.01). Therefore, the driver should also be trained in the driving behavior in hazardous events while improving the driver’s hazard perception ability.
